# Exploring the mineral–water interface: reduction and reaction kinetics of single hematite (α-Fe_2_O_3_) nanoparticles

**DOI:** 10.1039/c5sc03678j

**Published:** 2015-11-18

**Authors:** K. Shimizu, K. Tschulik, R. G. Compton

**Affiliations:** a Department of Chemistry , Physical and Theoretical Chemistry Laboratory , Oxford University , South Parks Road , Oxford , OX1 3QZ , UK . Email: Richard.Compton@chem.ox.ac.uk ; Fax: +44 (0)1865 275 410 ; Tel: +44 (0)1865 275 957; b Nano-Electrochemistry – Center for Electrochemical Sciences , Faculty of Chemistry and Biochemistry , Ruhr-University Bochum , D-44780 Bochum , Germany

## Abstract

In spite of their natural and technological importance, the intrinsic electrochemical properties of hematite (α-Fe_2_O_3_) nanoparticles are not well understood. In particular, particle agglomeration, the presence of surface impurities, and/or inadequate proton concentrations are major obstacles to uncover the fundamental redox activities of minerals in solution. These are particularly problematic when samples are characterized in common electrochemical analyses such as cyclic voltammetry in which nanoparticles are immobilized on a stationary electrode. In this work, the intrinsic reaction kinetics and thermodynamics of individual hematite nanoparticles are investigated by particle impact chronoamperometry. The particle radius derived from the integrated area of spikes recorded in a chronoamperogram is in excellent agreement with electron microscopy results, indicating that the method provides a quantitative analysis of the reduction of the nanoparticles to the ferrous ion. A key finding is that the suspended individual nanoparticles undergo electrochemical reduction at potentials much more positive than those immobilized on a stationary electrode. The critical importance of the solid/water interface on nanoparticle activity is further illustrated by a kinetic model. It is found that the first electron transfer process is the rate determining step of the reductive dissolution of hematite nanoparticles, while the overall process is strongly affected by the interfacial proton concentration. This article highlights the effects of the interfacial proton and ferrous ion concentrations on the reductive dissolution of hematite nanoparticles and provides a highly effective method that can be readily applied to study a wide range of other mineral nanoparticles.

## Abstract 2

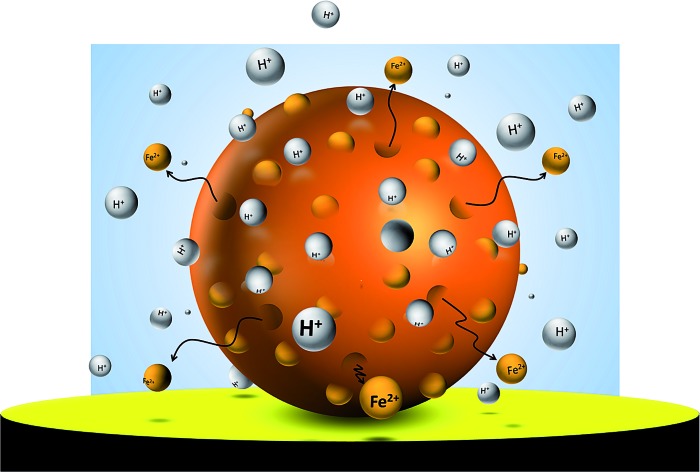
Here we show that particle impact chronoamperometry allows the quantitative electrochemical characterization of individual mineral nanoparticles with adequate proton concentrations. Through this approach, we extract the kinetics and thermodynamics of the reductive dissolution of single hematite (α-Fe_2_O_3_) nanoparticles.

## Introduction

Hematite nanoparticles form naturally in the soil through nucleation as the groundwater becomes supersaturated with clusters of the same mineral phase. They are an important source of bioavailable iron that is an essential nutrient for plants and microorganisms to prosper, to populate, and to diversify.^1^ They are also an effective adsorbent of various pollutants, which are then removed from or transported within the groundwater^2^,^3^ and the atmosphere.^4^ Nano-scaled hematite has also been intensively investigated in recent years in technological settings most notably as a photocatalyst in renewable hydrogen production *via* photoelectrochemical water splitting.^5^,^6^ In these respect, uncovering the intrinsic redox properties of hematite nanoparticles is of great interest in order to understand the (bio)geochemical iron cycle as well as to manipulate its surface for better catalytic activity.

Investigation of the redox reaction and/or electro-catalytic activities of hematite are often carried out on ensembles of nanoparticles, *i.e.* they are dried or directly grown on the surface of a working electrode. In such experimental conditions, maintaining the solution pH becomes important in order to avoid depletion of protons from the surface of modified electrode. This is because a concentration gradient affects the mineral's apparent redox activities and undesirable by-products can be formed. On the other hand, the use of pH buffers can alter physicochemical properties of hematite, as demonstrated by Lanzl *et al.*^7^ who reported an enhancement of the proton promoted dissolution of hematite at pH 2 by addition of oxalate. Moss *et al.*^8^ have also reported a significant increase in redox activity of nanoporous hematite when micro-molar concentration of phosphate is added to a perchlorate containing solution. This is because hematite is known to specifically adsorb various oxyanions *via* hydrogen bonding with the surface functional groups (*e.g.* –OH_2_, –OH, –O) and subsequently form a complex with iron atom by replacing surface water molecule.^9^,^10^ Such surface chemistry involving electrolytes can cast a shadow over the fundamental physicochemical behaviors of the mineral and should ideally be avoided.

Much intrinsic surface chemistry involving metal oxides, including the above mentioned processes, have been uncovered in parts by the potentiometric titration. This electrochemical analysis is used to determine the surface charges of mineral nanoparticles based on the amount of proton uptake during an acid–base titration.^11^ It is one of the most common approaches among researchers in surface and interfacial science to characterize mineral nanoparticles that are pre-dispersed in an aqueous solution. Many of interfacial processes involving hematite have also been probed by this measurement as well as the point of zero charge, the surface potential, and the formation constants of various surface complexes.^12^–^14^ This approach is however not ideal for an investigation of redox properties. The low concentration of supporting electrolytes often used for the experiment is also detrimental to study an electrochemical process because a redox reaction cannot be strictly diffusion controlled. Hence a new approach is desirable to examine intrinsic redox behavior of mineral nanoparticles.

An evaluation of redox properties of pristine metal oxide nanoparticles can be achieved by the recently developed particle-impact chronoamperometry as will be reported below. This electrochemical characterization technique depends on the random motion of a single nanoparticle suspended in an aqueous solution leading to collision with a stationary microelectrode. If the electrode potential is sufficient for an electrochemical reaction, electrons will be transferred between a particle and an electrode upon impact, which can be recorded as a spike in a chronoamperogram.^15^ Furthermore, when an electrochemical process is quantitative, the size of a nanoparticle can be determined from the charge obtained from the integrated area under a spike.^16^,^17^ This technique is, for example, employed in recent work by Tschulik *et al.*^18^,^19^ for an electrochemical sizing of magnetite nanoparticles. An accurate mean radius of Fe_3_O_4_, with respect to microscopic images of the same sample, was obtained from both anodic and cathodic impacts on a carbon fiber microelectrode in a dilute phosphate buffer solution.^18^ Reaction kinetics can also be investigated by conducting the particle-impact over a wide range of applied potentials as demonstrated by Haddou *et al.*^20^ in the oxidation of copper nanoparticles and by Cheng *et al.*^21^ in the reduction of indigo nanoparticles.

Herein particle impact chronoamperometry is employed to study the reductive dissolution of individual hematite nanoparticles in a dilute KCl solution at pH 2. This electrolyte represents the simplest aqueous environment to allow the pristine mineral/water interface to be analyzed. In addition, the interfacial chemistry under the given experimental condition is very well understood. For instance, under acidic pH, the chloride ion is the predominant species in the hematite/aqueous solution interface to counter balance the positively charged mineral surface,^13^ and hematite has no known surface chemistry involving this anion.^14^ Proton promoted chemical dissolution of hematite is negligible within the time scale of the particle-impact experiment because of the slow rate of the reaction.^7^ To the best of our knowledge, this is the first attempt to investigate the electrochemical reduction of pristine single hematite nanoparticles suspended in an aqueous solution. Successful application of this technique will open an entirely new way of characterizing the mineral/water interface.

## Experimental

### Chemicals and materials

All chemicals were used as received. Potassium chloride was obtained from Sigma Aldrich (≥99.0%, Steinheim, Germany). Analytical reagent grade hydrochloric acid was purchased from Fisher (∼37%, Loughborough, UK). The concentration of diluted acid was reassured by titration against Tris buffer. Ultrapure water used throughout this work was filtered through Millipore SimPak® 1 purification pack (lot# F5BA50456). Oxygen free N_2_ gas (99.998%, BOC Gases plc, Guildford, UK) was humidified by passing through traps containing ultrapure water and 0.1 M NaOH. Electrolyte solution was prepared by mixing 20 mM KCl with appropriate amount of dilute hydrochloric acid solution of the same ionic strength. The pH of the solution was monitored by a PH 213 microprocessor pH meter (HANNA Instruments, Leighton Buzzard, UK) and a pH electrode (HI 1131, HANNA Instruments) that was calibrated daily. Glassy carbon (3 mm in diameter), gold (2 mm in diameter), and gold micro (11 μm in diameter) electrodes were purchased from Bioanalytical Systems Inc. (Stareton, UK), and a Ag/AgCl (saturated KCl) reference electrode was obtained from ALS Co. Ltd. (Tokyo, Japan). All electrochemical measurements were conducted using a Metrohm μAutolab II potentiostat (Utrecht, the Netherland) with Nova (v.1.10.5) as an operating interface. The charge–potential plots were fitted using nonlinear least squares regression that employed the Levenberg–Marquardt algorithm. This calculation was carried out using Origin Lab Pro 2015 (OriginLab Co., Northampton, USA).

### Hematite synthesis and characterization

**Fig. 1:**
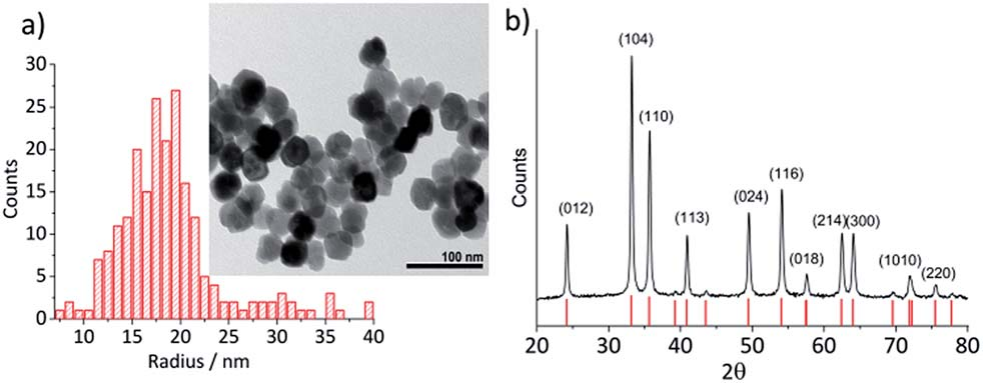
(a) A TEM image of hematite nanoparticles prepared by forced hydrolysis and a histogram illustrating a distribution of particle radius based on 215 individuals with the mean radius of 18.0 ± 2.5 nm. (b) The powder XRD spectrum of hematite nanoparticles. Peak positions are in good agreement with the reference patterns, PDF#072-0469,^25^ which is shown at the bottom of the figure. Miller indexes are assigned to each peak according to Wang *et al.*^24^ for α-Fe_2_O_3_ prepared under a similar condition to this study.

Hematite nanoparticles used in this study was prepared in-house according to the procedure described in ref. 22. Briefly, it was synthesized by forced hydrolysis by adding a 0.72 M FeCl_3_ solution dropwise to a vigorously stirred 3 mM HCl solution that was preheated to 100 °C. After maturing the mixture in an oven at 100 °C for 7 days, it was dialyzed until the resistivity of the supernatant exceeded 10^6^ Ω cm. The resulting products were then transferred to a Nalgene® bottle and kept in a suspension form. The size and morphology of the mineral particles were checked by transmission electron microscopy (TEM) using a Jeol JEM 1230 microscope equipped with a digital multi-scan camera (Gata MSC 600CW). Some particles shown in the TEM image (Fig. 1a) are found to have a distinctive polygonal shape, an expected shape of hematite that is synthesized in the hydrothermal method,^23^,^24^ while morphologies of most others cannot be clearly identified either as polyhedral or spherical. The particle size measured over 215 particles is found to have the mean radius of 18.0 ± 2.5 nm (Fig. 1a). Structural purity of hematite was confirmed by a Bruker d8 Advance X-ray diffractometer (XRD) using Cu Kα radiation and scanning angle swept from 20° to 80° at a rate of 0.008° s^–1^. The XRD spectrum of the synthesized nanoparticles (Fig. 1b) is in good agreement with the reference patterns for this minerals (PDF#072-0469) given by Blake *et al.*^25^ as well as that of synthetic hematite reported previously by Wang, *et al.*^24^ No detectable residue of chloride ion from the synthesis was found by X-ray photoelectron spectroscopic analysis conducted by a Kratos Axis Ultra electron spectrometer equipped with a delay line detector and a monochromated Al Kα source operated at 150 W (result not shown).

### Electrochemical analysis

All electrochemical analyses are carried out in a conventional three-electrode setup inside of a Faraday cage at 25 ± 0.2 °C under dark condition. Prior to experiment, electrolyte solution was deoxygenated by bubbling a rapid stream of N_2_ through it. Working electrodes were polished on aqueous slurries of 1, 0.3, and 0.05 μm alumina for 3 to 5 min each in descending order of size. Alumina residue was removed from the electrode by rinsing with plenty of ultrapure water and wiping on a dry, plain polishing cloth.

### Cyclic voltammetric analysis

The working electrode was modified by drying a 2 μL droplet of a dilute hematite suspension under ambient conditions for at least 30 min. Once dried, the modified electrode was submerged in a deoxygenated electrolyte solution with a desired pH for 10 min before the potential was swept between 0.6 V and –0.6 V *vs.* Ag/AgCl (–0.8 V for a glassy carbon electrode) at a scan rate of 0.01 V s^–1^.

### Particle-impact chronoamperometry

Hematite nanoparticles were dispersed in deoxygenated 20 mM KCl (pH 2.1) in an ultrasonic bath for 5 min. Chronoamperograms were recorded at a gold microelectrode for 50 s with a measurement interval time of 0.5 ms at potentials ranging from –0.5 to 0.7 V *vs.* Ag/AgCl. A minimum of 30 chronoamperograms were collected at each potential. Identification and analysis of spikes were carried out by Signal Counter software developed by Dr Dario Omanović, Ruđjer Bošković Institute, Zagreb, Croatia.^18^

## Results and discussion

In this section, the cyclic voltammetric analysis of the reduction of hematite nanoparticles immobilized on a stationary macro-electrode is first discussed in order to elucidate effects of proton depletion and particle agglomeration. This analysis was also necessary to determine the initial potential range which particle impact chronoamperometry would be conducted. Subsequently, the reduction of the individual nanoparticles is addressed, and the results are interpreted using a kinetic model provided hereinafter.

**Fig. 2:**
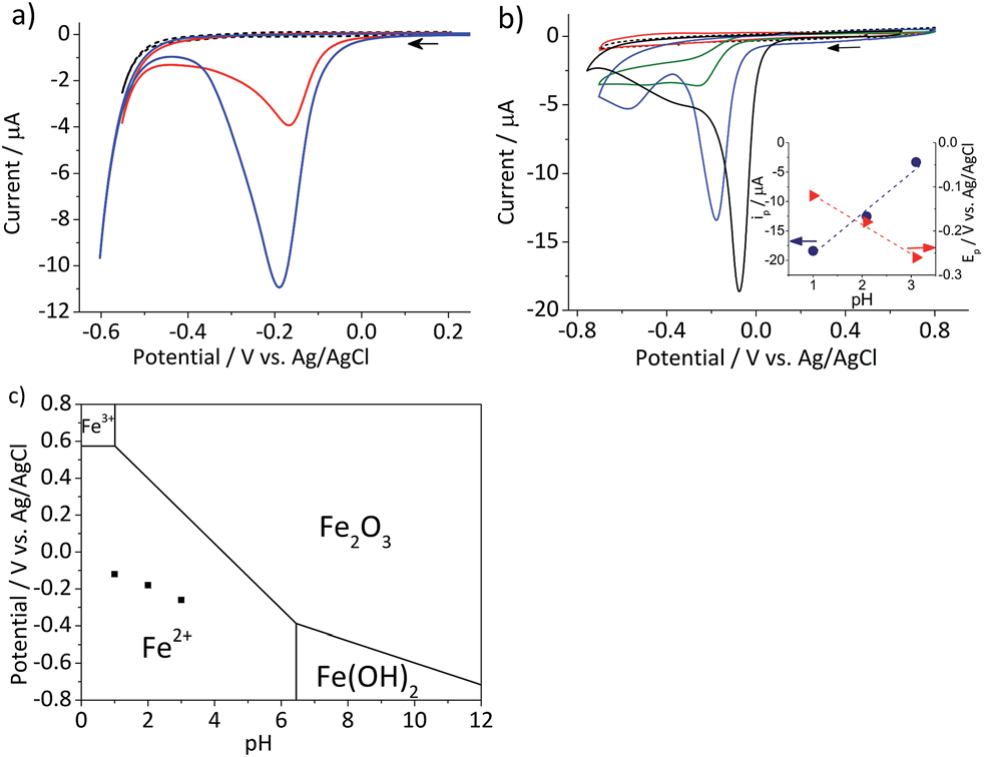
(a) Characteristic cyclic voltammograms of gold electrodes modified with 2.8 × 10^–5^ g cm^–2^ (red) and 5.5 × 10^–5^ g cm^–2^ hematite nanoparticles (blue) and an unmodified gold electrode (black dash) recorded at a scan rate of 0.01 V s^–1^ in deoxygenated 20 mM KCl at pH 2.1 adjusted by addition of appropriate amounts of 20 mM HCl. (b) Cyclic voltammograms of glassy carbon electrodes modified with 2.4 × 10^–5^ g cm^–2^ hematite recorded at a scan rate of 0.01 V s^–1^ in deoxygenated 20 mM KCl solutions at pH 4.4 (red), 3.1, (green), and 2.1 (blue). A cyclic voltammogram at pH 1 (black solid line) was collected in 0.1 M HCl and that of a bare glassy carbon electrode (black dash) was collected at pH 2.1. Arrows indicate the scan direction. The inset shows the plots of the peak current, *i*_p_, (blue circle) and the peak potential, *E*_p_, (red triangle) as a function of pH. (c) The Pourbaix diagram of iron^27^ at the ambient conditions, while black squares indicate the peak potentials observed in (b).

Cyclic voltammetry was performed on the mineral immobilized on a gold macro-electrode in deoxygenated 20 mM KCl solution at pH 2.1. This experiment was conducted with two different hematite loadings, 2.8 × 10^–5^ g cm^–2^ (equivalent to an average of 1.6 monolayers of hematite particles on the electrode) and 5.5 × 10^–5^ g cm^–2^ (3.3 monolayers). Typical voltammograms of hematite modified electrodes shown in Fig. 2a have a single reduction peak at around –0.19 V *vs.* Ag/AgCl, and the signal scales with the hematite loading. The currents at around the cathodic switching potential is attributable to hydrogen evolution reaction occurring at the gold working electrode.^26^

Given the acidic nature of the solution and considering the Pourbaix diagram of iron (Fig. 2c), hematite is expected to undergo 2-electron reduction as shown below:

1 Fe_2_O_3_ + 6H^+^ + 2e^–^ → 2Fe^2+^ + 3H_2_O

The dependency of hematite reduction on the surface proton concentration was further investigated by performing cyclic voltammetry in deoxygenated 20 mM KCl at pH between 4.4 and 1 with the hematite nanoparticles immobilized on a glassy carbon electrode. The amount of hematite used (2.4 × 10^–5^ g cm^–2^, an average of 1.5 monolayers) was the same for all pHs. The ionic strength of the electrolytic solution was maintained at 20 mM except for the pH 1 solution, in which the ionic strength was 0.1 M. The reduction of hematite immobilized on a stationary electrode was found to take place at the solution pH value below 4 as shown in Fig. 2b. The dominant voltammetric peak, which appears at the more positive potential in the voltammogram, has a peak shape consisted with the reduction of a surface immobilized nanoparticles.^28^ The secondary peak is attributable to oxygen reduction reaction on the bases of “fingerprinting” experiment under an oxygen saturated solutions (result not shown). As generation of oxygen under the given experimental conditions is not thermodynamically plausible, it is likely trapped or adsorbed while nanoparticles are casted onto a stationary electrode and thereafter released as the hematite is dissolved.

The correlation between the primary peak potentials and currents with acidity shown in Fig. 2b inset is attributable to the surface proton concentration. The peak potential is found to shift cathodically with respect to the solution pH at a slope of –0.066 V per pH. This value is considerably smaller than the expected slope of –0.177 V per pH calculated for eqn (1) by the Nernst equation:2
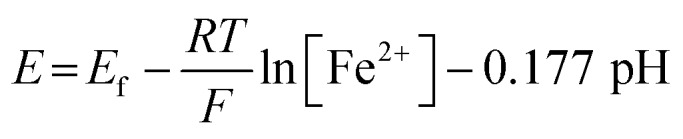
where *E* is the electrode potential and *E*_f_ is the formal potential of eqn (1). *R*, *T*, and *F* denote the gas constant (= 8.314 J mol^–1^ K^–1^), temperature (= 298.15 K), and the Faraday constant (= 96 485.3 C mol^–1^). Overlaying the peak potentials on the Pourbaix diagram (Fig. 2c); it is found that the local pH near the electrode surface is above 5. Under such conditions, it is conceivable that hematite is forced to undergo a reduction process that involves fewer protons than in eqn (1), producing secondary iron oxides such as ferrous hydroxide on the surface (Fig. 2c). Products like Fe(OH)_2_ have a poor solubility in water (log *K*_sp_ = –16.3) and passivate the hematite surface from further reduction. Furthermore, the immobilized nanoparticles are likely agglomerated on the electrode surface, and the cluster formation can lead to incomplete stripping of nanoparticles.^28^ Agglomeration is evident from the integrated charges under the cathodic peaks found in Fig. 2a. The charge for the high hematite loading (5.5 × 10^–5^ g cm^–2^) in Fig. 2a is 0.18 mC that is considerably less than the value calculated from the amount used, which should correspond to 2.1 mC. Similarly, the lower hematite loading showed a reduction peak area of only 0.04 mC, while 1.0 mC was expected. Even at pH 1 (Fig. 2b), the maximum charge obtained is only 0.45 mC, that is less than 25% of the expected value. Based on these results, it can be concluded that the interpretation of the voltammograms is difficult and ambiguous because of the complexity of interfacial (electro)chemistry at the surface of nanoparticles immobilized on a stationary electrode, and hence it is not an ideal way to characterize hematite/water interface.

**Fig. 3:**
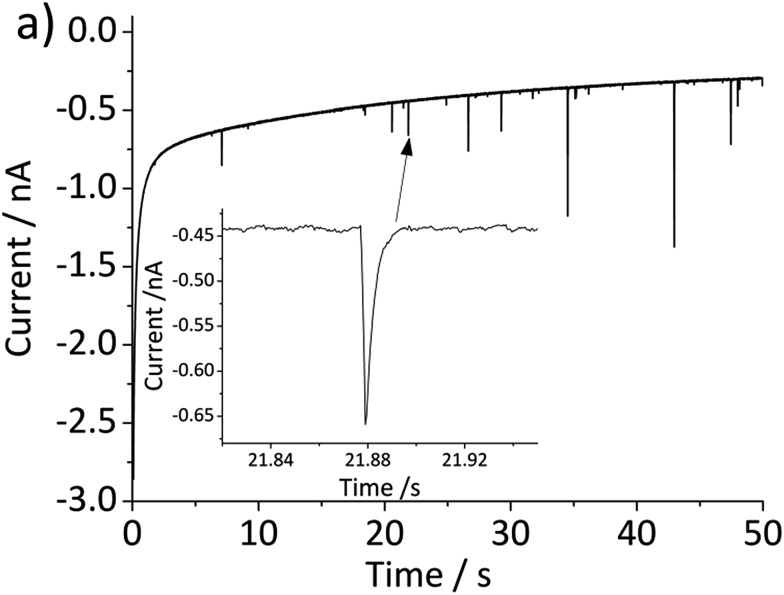
Characteristic chronoamperogram recorded at a gold microelectrode in deoxygenated 20 mM KCl with pre-dispersed hematite nanoparticles at pH 2.1 and at an applied potential of –0.4 V *vs.* Ag/AgCl. The inset provides an enlarged view of the current–time profiles illustrating the faradaic reduction of nanoparticles upon collision with the working electrode as spikes.

The above problem is circumvented by using the single particle impact approach owing to the greatly improved mass transport to individual nanoparticles (convergent diffusion) instead of the linear diffusion to an ensemble of nanoparticles immobilized on a surface. The improved mass transport of protons to the nanoparticle surface makes the electrochemical process less prone to proton depletion and prevents formation of any secondary iron oxides. Furthermore, it enables the quantitative dissolution of single hematite nanoparticles. In this study, hematite nanoparticles are pre-dispersed in a well deoxygenated 20 mM KCl at pH 2.1, and a minimum of 30 chronoamperograms are collected at each electrode potential. For applied potentials between –0.5 V and 0.45 V *vs.* Ag/AgCl, negative current spikes are observed from faradaic reduction of individual hematite nanoparticles. A characteristic chronoamperogram that was collected at –0.4 V *vs.* Ag/AgCl is shown in Fig. 3.

**Fig. 4:**
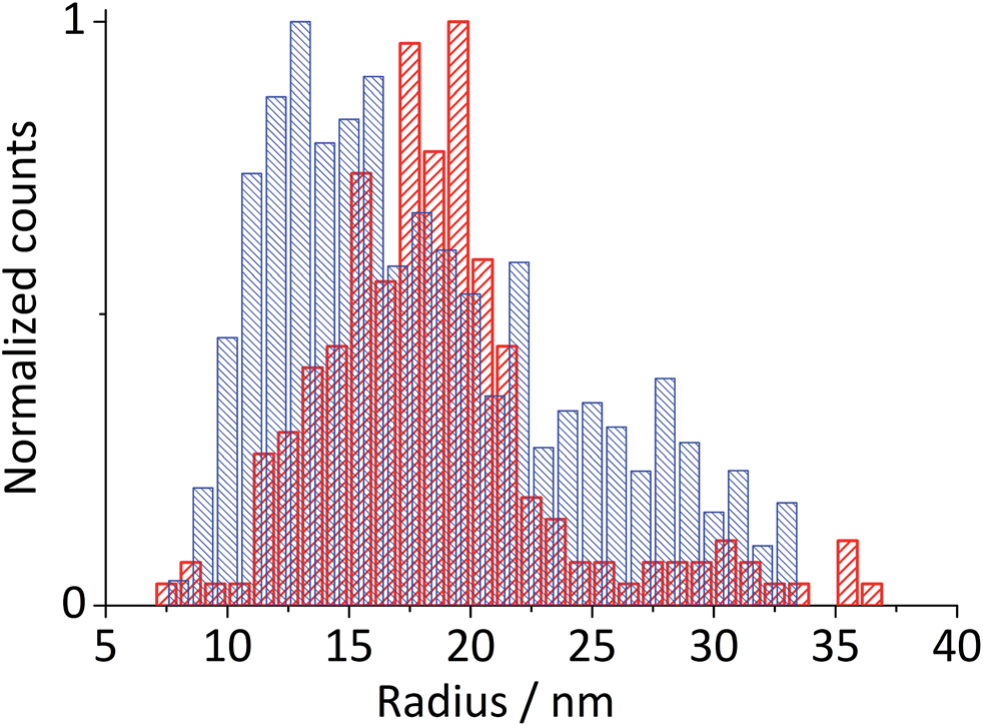
Histogram of particle radius determined from spikes obtained during chronoamperometry at –0.4 V *vs.* Ag/AgCl and corrected for the diffusion coefficient (blue, dense mesh) and from TEM (red, medium mesh). The mean value of 15.2 ± 1.8 nm is determined from a log-normal distribution curve of radius resulting from the particle impact.

The charge passed during an impact, *Q*, is obtained by integrating the area under a spike, and the radius of a particle is calculated using the following equation^18^3
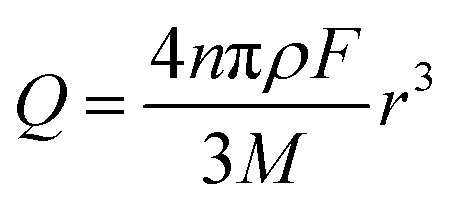
where *n* is the number of electrons transferred per Fe_2_O_3_ unit (= 2), *ρ* is the density of hematite (= 5.3 g cm^–3^), *M* is the molecular mass of Fe_2_O_3_ (= 159.678 g mol^–1^), and *r* the particle radius. Fig. 4 compares the histogram of the radius calculated from 713 impact spikes recorded at –0.4 V *vs.* Ag/AgCl with that from TEM images (sample size of 215). Note that the size obtained from the particle impact experiments is skewed to the left because it is limited by the minimum detectable spike (the root-mean-square noise of the experimental setup is about 3 pA). Also, it may be related to the fact that the smaller particles have a greater diffusion rate.^29^ However this can be corrected by applying the Einstein–Stokes equation to estimate the nanoparticle diffusion coefficient, *D*, as a function of size and normalizing the data by dividing by *D*.^30^ Nevertheless, the mean of the lognormal distribution of the particle radius shown in the figure is 15.2 ± 1.8 nm, which is slightly smaller than the mean radius of particles captured in TEM images (18.0 ± 2.5 nm). This small difference maybe attributable to the quasi-spherical shape of the hematite nanoparticle as captured in the TEM images (Fig. 1a) leading eqn (3) to underestimate the radius.^31^

**Fig. 5:**
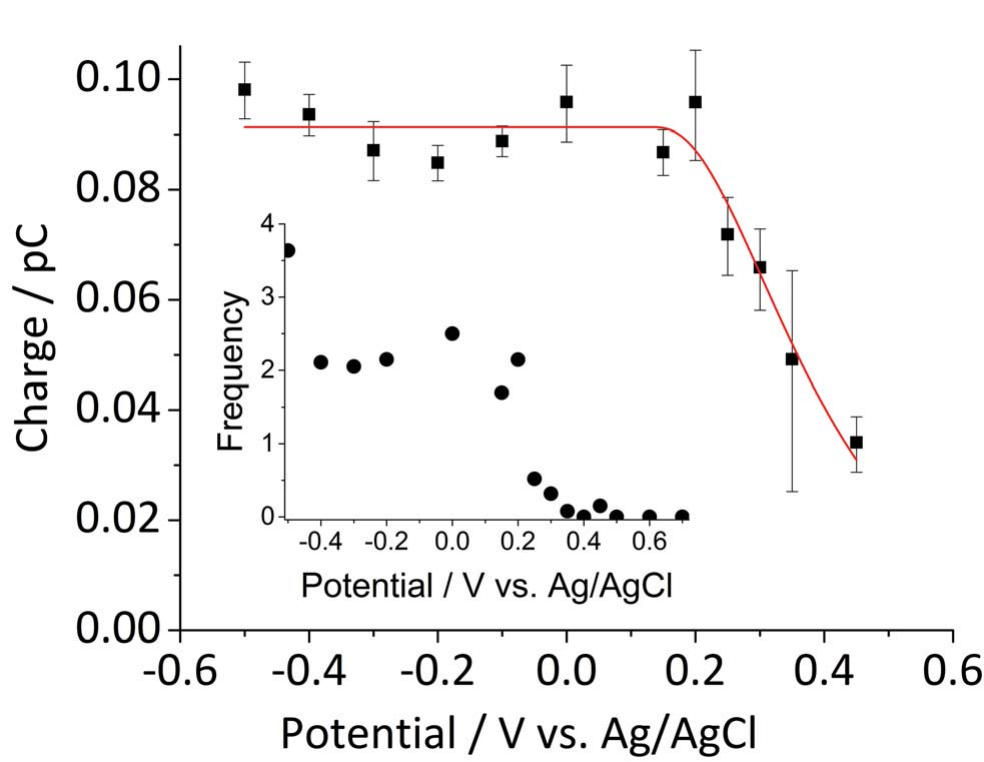
Log normal average reductive charges for individual hematite nanoparticles in deoxygenated 20 mM KCl at pH 2.1 as a function of the applied potential (black square). Error bars indicate the standard error of the mean. The result of nonlinear curve fitting given by eqn (4) is shown as red line. Parameters extracted from the fit are *r*_i_ = 15.0 ± 0.1 nm, *E*_1/2_ = 0.377 ± 0.017 V *vs.* Ag/AgCl, (*n*′ + *α*) = 0.17 ± 0.03, and 
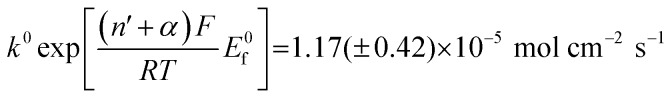
. The inset shows the impact frequency, *i.e.* the number of spikes observed per chronoamperogram, as a function of the electrode potential.

Fig. 5 shows the log-normal average of integrated charges under spikes as a function of electrode potential. It remains relatively constant from –0.5 V *vs.* Ag/AgCl to 0.25 V *vs.* Ag/AgCl showing a quantitative reduction of the impacting hematite nanoparticles at these potentials. Upon application of more positive potentials, a gradual decrease of the average impact charge is observed. This is indicative of that the driving force is insufficient to ensure a fast enough electron transfer for quantitative reductive dissolution of a single nanoparticle during its impact. This is also reflected in the average number of spikes observed per chronoamperometric measurement (Fig. 5 inset) that shows the frequency drops drastically at the same potential as the charge starts to decrease, and no spike is recorded at potentials more positive than 0.45 V *vs.* Ag/AgCl. This potential is close to the standard reduction potential of free ferric/ferrous ion pair, 
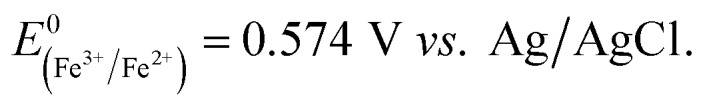
 This observation uncovers the previously unknown electrochemical activity of the pristine hematite nanoparticles. In particular, the very large difference in potential seen between single nanoparticles and the ensembles studied *via* cyclic voltammetry is noteworthy. The impact behavior is robust and much less sensitive to the proton depletion effect. Moreover, the reduction potential for the Fe_2_O_3_ nanoparticle is close to that expected on the basis of the Pourbaix diagram (Fig. 2c).

We next consider the kinetics of the nanoparticle dissolution. Kinetic models for the irreversible electrochemical dissolution have been described in detail elsewhere.^20^ The expression for potential dependent reductive dissolution of individual hematite nanoparticles is given as:^20^,^21^
4
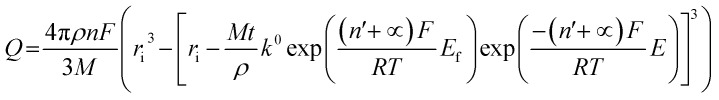
where *r*_i_ is the initial radius of nanoparticle, *k*^0^ is the electrochemical rate constant in mol cm^–2^ s^–1^, *n*′ is the number of electrons transferred prior to the rate determining step, *α* is the transfer coefficient, *E*_f_ denotes the formal potential, and *t* is the duration of time which particle is within the tunnelling distance from the electrode surface where electron transfer can occur.^32^ The time parameter is governed by the random motion of a particle and independent of the electrode potential. The impact duration of 10 ms is found as the average of base width of all observed spikes during this experiment, thus it is used for the kinetic model fitting. The “half wave” potential for particle impact is defined as the potential at which the average charge passed per spike is half of the average charge passed under diffusion limited condition, it can be expressed as:^20^5
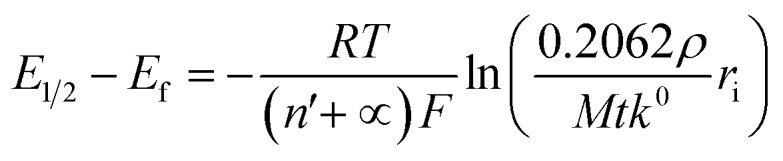



The nonlinear regression curve fitting using eqn (4) was carried out for particle radius, *r*_i_, the combined kinetic parameter, 
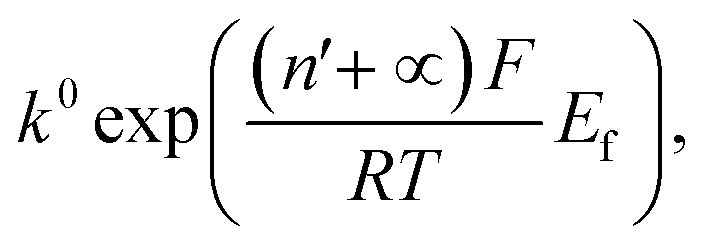
 and the effective transfer coefficient, (*n*′ + *α*). Eqn (4) fits the experimental result well (Fig. 5, a red solid line) with the *χ*^2^ value in the order of 10^–5^. The particle radius obtained from the fit is 15.0 ± 0.1 nm that is within the errors of previous result using eqn (3). The (*n*′ + *α*) value is found at 0.17 ± 0.03, indicating that the rate-determining step of the reductive dissolution of hematite nanoparticles is the first electron transfer (*n*′ = 0). This observation is in line with previous discussion and the “half wave” potential is found at 0.377 ± 0.017 V *vs.* Ag/AgCl, which is significantly more positive than the reduction of immobilized hematite nanoparticles shown in Fig. 2. The combined kinetic parameter is found at 1.17 (±0.42) × 10^–5^ mol cm^–2^ s^–1^.

Based on these findings, the mechanism for reductive dissolution of hematite nanoparticle can be postulated as being driven by the reduction of Fe(iii) to Fe(ii). Thereupon, ferrous ion is released from the surface and diffuses away. This process is particularly promoted for suspended nanoparticles because of the essentially undepleted interfacial proton concentration and the radial diffusion of ferrous ion away from the particle surface.^28^,^33^ This is evident in a comparison with the cyclic voltammetric study, in which only a fraction of hematite nanoparticles immobilized on a stationary electrode is reduced.

## Conclusions

This study addresses the reductive dissolution of hematite nanoparticle suspended in an acidic aqueous solution probed by particle impact chronoamperometry. Single hematite nanoparticles are electrochemically reduced when their random, Brownian motion results in a collision with the surface of a stationary microelectrode. The reduction of nanoparticles pre-dispersed in an electrolytic solution takes advantage of the efficient diffusion of protons to the particle/water interface, ensuring an ample supply of the cation to facilitate a fast and quantitative reduction at a small overpotential. This is contrary to the conventional drop-cast approach in which an incomplete reduction of hematite caused by proton deficiency at the electrode/water interface causes strongly distorted cyclic voltammograms. The kinetic analysis uncovers that the rate determining step is the first charge transfer process. The result also indicates the low surface concentration of the ferrous ion due to rapid diffusion away from the particle surface. Such interfacial processes have resulted in the significantly more positive reduction potential observed by the particle impact than by cyclic voltammetric analysis. This investigation demonstrates a promising application of particle impact chronoamperometry for the electrochemical characterization of mineral nanoparticles by quantifying the reduction of pristine hematite nanoparticles.
